# Identification and Validation of a New Male Sex-Specific ISSR Marker in Pointed Gourd (*Trichosanthes dioica* Roxb.)

**DOI:** 10.1155/2014/216896

**Published:** 2014-11-05

**Authors:** Sinchan Adhikari, Soumen Saha, Tapas Kumar Bandyopadhyay, Parthadeb Ghosh

**Affiliations:** ^1^Cytogenetics & Plant Breeding Section, Plant Biotechnology Research Unit, Department of Botany, University of Kalyani, Kalyani, Nadia, West Bengal 741235, India; ^2^Department of Molecular Biology & Biotechnology, University of Kalyani, Kalyani, Nadia, West Bengal 741235, India

## Abstract

The aim of the present study was to develop a genetic sex marker for the pointed gourd (*Trichosanthes dioica* Roxb.) to allow gender determination at any stage in the life cycle. Screening of genomic DNA with intersimple sequence repeat (ISSR) primers was used to discover sex-specific touch-down polymerase chain reaction (Td-PCR) amplification products. Using pooled DNA from male and female genotypes and 42 ISSR primers, a putative male specific marker (~550 bp) was identified. DNA marker specific to male is an indication of existence of nonepigenetic factors involved in gender development in pointed gourd. The ISSR technique has proved to be a reliable technique in gender determination of pointed gourd genotypes at the seedling phenophase. The sex marker developed here could also be used as a starting material towards sequence characterization of sex linked genes for better understanding the developmental as well as evolutionary pathways in sexual dimorphism.

## 1. Introduction

Dioecism accompanied by sex chromosome dimorphism is common in animals but less prevalent in plants. In a minority of dioecious plants, sex determination depends on sex chromosomes, usually an XY system, in which males are heterogametic (XY) and females are homogametic (XX) [1–3]. There are two types of sex chromosomes [2]: homomorphic sex chromosomes, in which the sex chromosomes are morphologically indistinguishable from autosomes and heteromorphic sex chromosomes, which can be discriminated in cytological analyses. Heteromorphic sex chromosomes have been reported in several families (e.g.,* Cannabis* and* Humulus*, Cannabinaceae;* Silene*, Caryophyllaceae;* Rumex*, Polygonaceae) [2], but our understanding of their evolution and genetics is still relatively poor. The presence of dioecious forms in a number of Cucurbitaceous genera makes it an interesting model family to study sexual dimorphism, but the mechanism of sex determination has been studied in only a few such as* Coccinia* and* Bryonia* [4, 5].

Pointed gourd (*Trichosanthes dioica* Roxb.) of the family Cucurbitaceae is a perennial, dioecious crop extensively cultivated in tropical and subtropical regions around the world. In India, it is largely grown as a nutritious vegetable in the northern plains from Uttar Pradesh, West Bengal, Bihar, Orissa, to Assam. Breeding programmes in pointed gourd have several constraints such as poor germination, vegetative means of propagation, and dioecy. The plant strictly maintains the sexual phenotypes of male and female indicating clear genetic difference between both sexes. This clear differentiation of sexual phenotype, combined with its perennial nature, an increasing economic importance of the crop, and recent interest in breeding improved cultivars, makes the species attractive for the study of different aspects of sex determination. A reliable molecular strategy for the early identification of sex in* T. dioica* has been a priority in breeding programs in order to increase their economic potential and better understanding the developmental as well as evolutionary pathways of dimorphism.

The use of polymerase chain reaction (PCR)-based molecular markers, useful tools for determining genotypes in plant breeding, has now made it possible to select superior or exclude unwanted individuals at the DNA level. Specific genes or markers underlying interesting traits were mapped and identified via molecular markers and can be used to increase the efficiency and precision by selecting individuals that have the desired genetic traits at an early stage [6]. Several molecular markers such as RAPD, AFLP, and microsatellites have been used for early determination of sex in many agronomically important plants before they enter the reproductive stage [7–13]. Since 1994, a new molecular marker technique developed by Zietkiewicz et al. [14] called intersimple sequence repeat (ISSR) has been available. The technique involves the use of a microsatellite core unit bearing oligonucleotide primers, usually 16–25 bp long, nonanchored or anchored at the 5′ or 3′ end with 1–4 degenerate nucleotides. ISSR can be a rapid and easy technique for identifying gender, because it overcomes many of the technical limitations of RAPD and AFLP due to its high reproducibility and simplicity [15].

The aim of the present study was to develop a genetic sex marker for* T. dioica* to allow gender determination at any stage in its life cycle. The importance of the findings in the early identification of sex as well as the possible implications in the understanding the molecular basis of sex determination in* T. dioica* is also discussed.

## 2. Materials and Methods

### 2.1. Site Description

West Bengal (20° 31′–27°12′ North and 85° 50′–89° 52′ East), a state in the eastern zone of India, is bounded on the north by Sikkim and Bhutan, on the east by Assam and Bangladesh, on the south by the Bay of Bengal and on the west by Orissa, Bihar, and Nepal. West Bengal covers a geographical area of 88,752 sq. km, which constitutes about 2.7% of the total land area of India. The diversified landforms, soils, and temperatures provide a complex topography of West Bengal with five agroclimatic zones: Hill Zone (2.4–8 lakh ha), Terai Zone (2.149 lakh ha), Old Alluvial Zone (17.537 lakh ha), New Alluvial Zone (15.304 lakh ha), Red and Laterite Zone (24.842 lakh ha), and Coastal and Saline Zone (14.569 lakh ha).

### 2.2. Plant Materials

**Figure 1:**
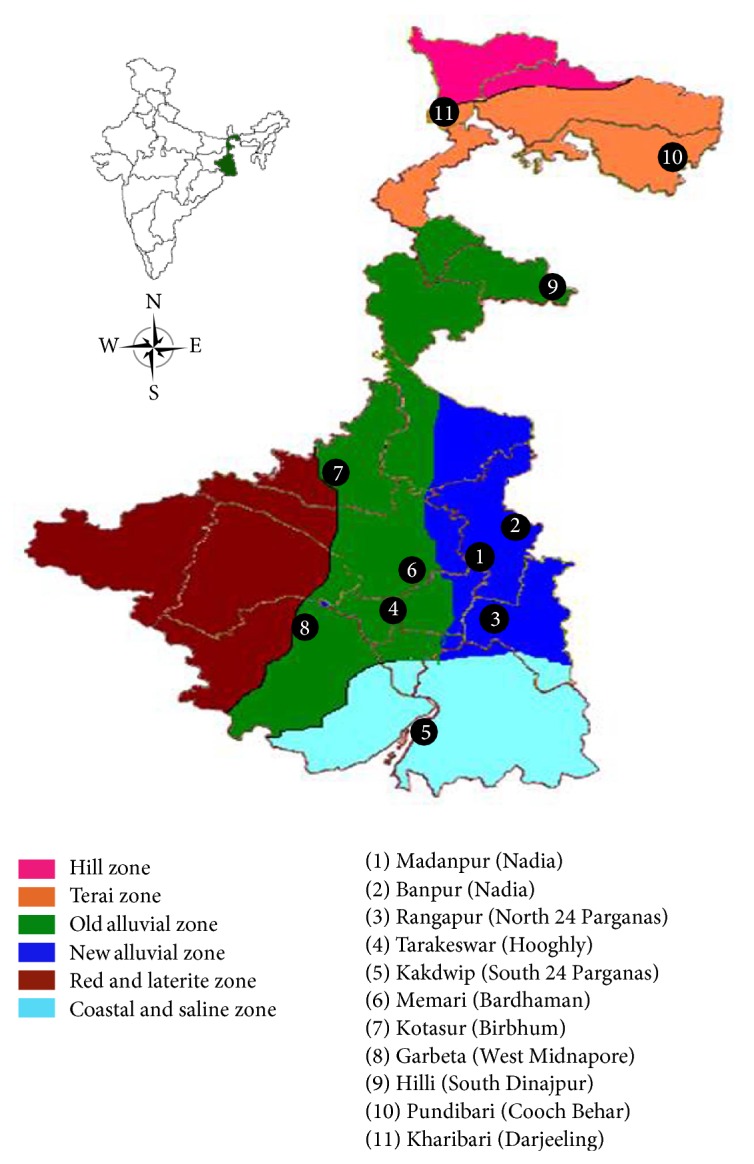
Agroclimatic zones of West Bengal showing localities of collection of pointed gourd accessions. Collection sites are marked with a circle and numbered from 1 to 11.

Male and female individuals of twenty cultivated accessions of pointed gourd were procured randomly from isolated areas of four different agroclimatic zones of West Bengal, India (Figure 1; Table 1). Immature leaf materials collected from sample of adult pistilate and staminate plants after complete observation of flower types were used for DNA extraction. The leaf materials were stored at −80°C prior to DNA extraction.

### 2.3. DNA Extraction

Total genomic DNA was isolated separately from 100 mg of leaf tissues from male and female individuals of the twenty accessions each according to our established protocol [16]. Concentration of the all DNA samples was readjusted so that each sample contained 30 ng/*μ*L genomic DNAs after spectrophotometer and agarose gel electrophoresis analyses.

### 2.4. Bulk Segregant Analysis (BSA)

Bulked segregant analysis [17] is a method used for rapidly identifying markers linked to any specific gene or genomic region. Two bulked DNA samples are generated from a segregating population from a single cross. Each pool or bulk contains individuals that are identical for a particular trait or genomic region but arbitrary in all unlinked regions. The two bulks are therefore genetically dissimilar in the selected region but seemingly heterozygous in all other regions [17]. Two bulk DNA samples (male and female) were prepared separately by pooling an equal amount of DNA from male and female individuals of the twenty accessions each and amplified with 42 primers. A DNA marker present in the corresponding male and female bulk and absent in the alternate sex bulk was considered as a potential sex-linked marker. BSA was used to screen each individual of known sex independently to identify the sex specificity of the marker.

### 2.5. Touch-Down Polymerase Chain Reactions

In the present study a touch-down polymerase chain reaction (Td-PCR) approach was used to amplify pointed gourd DNA samples. A total of 42, both 3′ anchored and unanchored 14–19 mer ISSR primers (Bangalore Genei, Bangalore, India), were screened. Each Td-PCR was performed in volumes of 25 *μ*L containing 2.5 *μ*L of 10x assay buffer (100 mM Tris–Cl; pH 8.3, 500 mM KCl, 15 mM MgCl_2_), 200 *μ*M dNTP mix (Bangalore Genei, Bangalore, India), 10 picomoles of primer, 1.0 unit of Taq DNA polymerase (Bangalore Genei), and 30 ng of template DNA. Touch-down polymerase chain reactions were carried out in a DNA Thermal Cycler (Eppendorf, Hamburg, Germany) with the following amplification profiles: 4 min hold at 94°C, followed by a 10-cycle pre-PCR consisting of 1 min at 94°C for denaturation, 1 min at 50 or 45°C for annealing, and 1 min 15 sec at 72°C for extension. The annealing temperature was reduced by 0.5°C per cycle for the first 10 cycles (touch-down cycles). Amplification of the targeted DNA templates continued for further 30 cycles at 40 or 45°C annealing temperature and ended with a final extension step at 72°C for 7 min. After Td-PCR completed, samples were stored at −20°C until agarose gel electrophoresis studies.

### 2.6. Agarose Gel Electrophoresis

Fifteen microliters of amplified Td-PCR products was mixed with 5 *μ*L loading buffer containing 0.25% (w/v) bromophenol blue, 0.25% (w/v) xylene cyanol FF, and 40% (w/v) sucrose. Samples were loaded into the wells of 2% (m/v) agarose gels. Agarose gels were prepared with 1X TAE buffer containing 40 mM Tris (pH 7.6), 20 mM acetic acid, and 1 mM EDTA. DNA samples were then electrophoresed at 4 V/cm at constant current for 8–16 h in the presence of 1X TAE buffer. After agarose gel electrophoreses completed DNA samples on the gels were visualized and photographed in Gel Documentation System (Uvitec, UK), all experiments were repeated at least three times with appropriate controls to ensure reproducibility and consistency.

## 3. Results and Discussion

**Figure 2:**
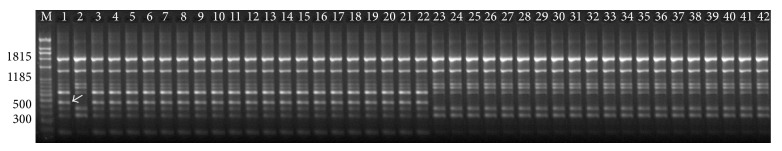
ISSR banding patterns of male and female plants of* Trichosanthes dioica* obtained with the primer ISSR-6 (5′-GACAGACAGACAGACA-3′). The arrow indicates the unique band of ~550 bp present in the male bulk. lane 1, bulk male; lane 2, bulk female; lanes 3–22, male individuals; lanes 23–42, female individuals; M denotes molecular mass marker (100 bp ladder).

Male and female DNA pools of twenty accessions of pointed gourd were screened with 42 ISSR primers for sex-specific polymorphisms and reproducibility. Since lengths, sequence, and annealing temperatures of primers used in the present study were quite different, the value selected for the annealing temperatures and other variables including the concentrations of Mg^2+^, dNTPs and template were determined empirically. The optimized Td-PCR produced repetitive results within and between PCRs and between templates obtained from each repeat of experiment. Of the 42 ISSR primers used to amplify the bulk DNA from male and female individuals, 21 primers gave a reproducible ISSR pattern (Table 2). Several DNA templates were amplified within the same PCR runs and between different PCR runs. The number of amplification products varied from 1 to 10, and the fragment sizes ranged from 100 to 2,000 bp. Reproducible results obtained from the Td-PCRs indicated that amplification of the nonspecific sequences was avoided. Of all the 42 primers tested, only one primer, ISSR-6, was found to show sex specificity in bulk analysis. Primer ISSR-6 (5′-GACAGACAGACAGACA-3′) produced a unique ~550 base-pair fragment in male bulk DNA, and this band was absent in female bulk DNA (Figure 2). To confirm this observation, the primer (ISSR-6) showing polymorphism for the sex type was used to retest the DNAs amplification with individual male and female samples from the twenty accessions. Interestingly, the unique ~550 bp fragment was present in all male individuals of the twenty accessions (Figure 2) and was completely absent in the respective female individuals tested.

Recent studies on sex-determining mechanisms in angiosperms have clearly revealed that a variety of sex-determining mechanisms have evolved which involve a number of different genetic and epigenetic parameters [18]. The most extreme type of sex determination system is found where highly specialized sex chromosomes are found; this type of system usually promotes dioecy in plants [19]. In the absence of genetic information on sex determination in dioecious plant, the use of molecular markers for discriminating between staminate and pistillate genotypes is worthwhile. PCR-based DNA technology has been proved to be a reliable strategy for detection of sex-associated markers in dioecious and bisexual taxa. In general, sex markers have been developed only rarely for plants with presumably autosomal sex determination, including* Atriplex garrettii* [20],* Salix viminalis* [21, 22], and* Uapaca kirkiana* [23]. It is clearly more likely to find sex-linked markers in cases where a sex chromosome system operates. Also, most of the known sex-linked markers are male associated, which reflects males being the heterogametic sex [1].

In earlier studies, three different molecular markers associated with sex expression in pointed gourd were screened using randomly amplified polymorphic DNA (RAPD) technique [24, 25]. However, they are not found to be reliable enough for detecting sex of pointed gourd genotypes from different geographical regions. Transcript profiling of unopened male and female floral buds of* T. dioica* has also been used in order to look for the differentially expressed unique and/or upregulated gene fragments associated with the sex expression; however, most of the fragments could not be annotated with any protein function reported till date [26]. Recently, sex-linked STS marker derived from ISSR fragment was developed for sex detection of* T. dioica* reflecting no significant homology with any structural gene sequences and did not carry any true open reading frame, probably indicating the paucity of information in this field [27].

Simple sequence repeats (SSRs) are abundantly present in the eukaryotic genomes making the amplification of delimited fragments with ISSR primers quick and easy. Moreover, ISSR markers lie in their linkage to SSR loci and are known to be linked to coding regions, so that ISSRs are likely to mark gene rich regions [28]. In the present study di, tri and tetra nucleotide SSR motifs GA, AG, GT, CA, AC CT, TG, CAC, GAG, CAA, CTC, GAA, and GACA were used. Out of these AG and GA motifs produced maximum scorable loci thus revealing more coverage of the genome, whereas (GACA)_4_, a nonanchored primer, developed a male linked marker. Amplification in the presence of nonanchored primers has also been called microsatellite-primed PCR or MP-PCR [29]. Such amplification does not require genome sequence information and leads to multilocus and highly polymorphous patterns [30].

The tetrameric GACA simple DNA sequence has received attention due to speculations on their relevance for sex determination in the heterogametic sex [31, 32]. This marker has been successfully applied in guppy fish [33] and mice [34]; however, scanty reports are available in plants. Gangopadhyay et al. [35] developed a female and hermaphrodite specific sex marker in papaya using (GACA)_4_. Sexual dimorphism was also revealed in betelvine using the tetrameric GACA sequence indicating the linkage of GACA repeat sequence with sex determining loci [36]. In contrary there are some instances where tetrameric GACA repeat (both anchored and nonanchored) was found to be unsuccessful in characterization of gender [37, 38].

## 4. Conclusions

Male linked ISSR-6_550_ marker is found to be reliable enough for detecting sex of pointed gourd genotypes from different geographical regions. This will facilitate screening of plants at the seedling stage and maintain an optimum sex ratio in plantations, as well as save time and costs in ongoing pointed gourd breeding programs. Moreover, sexual dimorphism is reported to be linked to many economically important traits such as disease resistance, leaf quality, and so forth. Exploring the relationship between sex-linked markers and desirable traits (especially to disease resistance, as pointed gourd is affected by many pathogens) in future will assist targeted genetic improvement. Further investigations are underway to sequence the male-specific gene fragment and then to compare it with other similar sequences in order to provide theoretical insights into the mechanism of sex differentiation in pointed gourd.

## Figures and Tables

**Table 1:** Pointed gourd accessions collected from different locations of West Bengal, India.

Accession name	Collection site	District	Latitude	Longitude	Altitude (feet)	Sex	
						Male (M)	Female (F)
Kajli	Madanpur	Nadia	23°1′0N	88°28′60E	29	M	F
Madanpur	Madanpur	Nadia	23°1′0N	88°28′60E	29	M	F
Banpur	Banpur	Nadia	23°26′60N	88°46′0E	42	M	F
Haribatkhali	Rangapur	North 24 Parganas	22°45′41N	88°22′19E	49	M	F
Haludkhali	Rangapur	North 24 Parganas	22°45′41N	88°22′19E	49	M	F
Pabda	Rangapur	North 24 Parganas	22°45′41N	88°22′19E	49	M	F
Dhapa	Rangapur	North 24 Parganas	22°45′41N	88°22′19E	49	M	F
Sada Patol	Rangapur	North 24 Parganas	22°45′41N	88°22′19E	49	M	F
Kali	Tarakeswar	Hooghly	22°53′60N	88°1′20E,	59	M	F
Sundari	Kakdwip	South 24 Parganas	21°52′60N	88°10′60E	3	M	F
Sandhyamoni	Kakdwip	South 24 Parganas	21°52′60N	88°10′60E	3	M	F
Damodar	Memari	Burdwan	23°11′60N	88°7′0E	78	M	F
Guli	Memari	Burdwan	23°11′60N	88°7′0E	78	M	F
Ghugut	Memari	Burdwan	23°11′60N	88°7′0E	78	M	F
Korda	Kotasur	Birbhum	23°56′60N	87°40′0E	173	M	F
Hijli	Garbeta	West Midnapore	22°49′60N	87°19′60E	177	M	F
Hilli	Hilli	South Dinajpur	25°13′0N	88°46′0E	82	M	F
Coochbehar local	Pundibari	Cooch Behar	26°15′0 N	89°30′0E	154	M	F
Kharibari	Kharibari	Darjeeling	26°21′0N	88°55′0E	190	M	F

**Table 2:** Reproducible ISSR primers, their sequences, and total number of amplified loci.

Primer code	Primer sequence (5′-3′)	Number of amplified loci
ISSR-1	(TG)_8_AT	3
ISSR^−^2	(GA)_8_C	5
ISSR-3	(GAA)_5_GA	6
ISSR-4	(GA)_6_CC	8
ISSR-5	(CTC)_4_TC	6
ISSR-6	(GACA)_4_	10
ISSR-7	(CA)_8_GT	3
ISSR-8	(CT)_8_GAC	1
ISSR-9	(CA)_6_AC	4
ISSR-10	(CT)_8_AC	2
ISSR-11	(CA)_8_A	3
ISSR-12	(CAA)_5_	3
ISSR-13	(AG)_8_A	5
ISSR-14	(CA)_7_G	3
ISSR-15	(GAG)_4_CA	6
ISSR-16	(AC)_9_	5
ISSR-17	(CAC)_6_	3
ISSR-18	(GT)_8_CA	2
ISSR-19	(AG)_7_C	5
ISSR-20	(GA)_8_A	6
ISSR-21	(AC)_8_T	4
